# Alcohol consumption: from biochemistry to behavior, health, and beyond

**DOI:** 10.3389/ftox.2026.1876876

**Published:** 2026-09-28

**Authors:** Evgeny Pechkovsky, Boris Kershengoltz, Valentin V. Vlassov, Vasiliy Vlassov, Andrey Turchinovich

**Affiliations:** 1 Research Institute of Molecular Biology and Biophysics, Federal State Budgetary Scientific Institution “Federal Research Center for Fundamental and Translational Medicine”, Novosibirsk, Russia; 2 Institute of Biological Problems of the Cryolithozone, Siberian Branch of the Russian Academy of Sciences, Yakutsk, Russia; 3 Institute of Chemical Biology and Fundamental Medicine SB RAS, Novosibirsk, Russia; 4 Higher School of Economics, Moscow, Russia; 5 Department of Medicine VI, University Medical Centre Mannheim, Heidelberg University, Mannheim, Germany; 6 Heidelberg Biolabs GmbH, Heidelberg, Germany

**Keywords:** Ethanol metabolism, *ADH1B*, *ALDH2*, *CYP2E1*, alcohol use disorder (AUD), alcohol-associated liver disease, metabolic dysfunction‐associated steatotic liver disease (MASLD)

## Abstract

Alcohol metabolism is a multi-step biochemical process driven primarily by hepatic oxidation of ethanol to acetaldehyde and then to acetate. Although this pathway protects the body from prolonged ethanol exposure, transient acetaldehyde accumulation generates substantial toxicity, and chronic alcohol intake activates auxiliary pathways that amplify oxidative stress, inflammation, and tissue injury. Both metabolic capacity and physiological response to alcohol vary widely between individuals, reflecting genetic and epigenetic variation in the alcohol- and aldehyde-dehydrogenase enzymes, the cytochrome P450 system, and modulating factors such as sex, age, body composition, nutrition, and overall health. In this review we consolidate 3 decades of biochemical, clinical, and epidemiological evidence into an integrated picture of alcohol’s effects on the liver, brain, and other organ systems, with attention to oxidative stress, neurochemistry, glycoconjugate biology, and the convergence of alcohol- and metabolic-driven liver disease. We also examine recent advances in the treatment of alcohol use disorder (AUD) - including incretin-based pharmacotherapies, ketamine-assisted therapy, focused ultrasound neuromodulation, exercise interventions, and combination drug regimens - and we critically evaluate ongoing controversies surrounding moderate drinking, resveratrol, and the use of heterogeneous non-drinker comparison groups in observational studies. Finally, we discuss the changing social context of drinking, the role of endogenous ethanol/acetaldehyde systems in stress resilience across species, and the implications of these insights for personalized prevention and risk-reduction strategies. We conclude by proposing an exposure-susceptibility-context model that integrates the principal determinants of alcohol-related harm.

## 1 Introduction

In contemporary society, alcohol remains embedded in many cultural practices, social rituals, workplace interactions, and interpersonal traditions (Mandelbaum, 1965; Castro et al., 2014; Savic et al., 2016; Sudhinaraset et al., 2016). Individuals routinely encounter alcohol in celebrations, business settings, and sanctioned rituals, where it can function as a tool for social bonding, group identity, and conformity to perceived norms (Sudhinaraset et al., 2016; Ratcliffe et al., 2019; Morris et al., 2020). Participation in alcohol-related customs may therefore contribute to psychological inclusion, particularly when drinking is associated with belonging or celebration (Savic et al., 2016; Ratcliffe et al., 2019; Raninen et al., 2021). Although social inclusion and supportive relationships are strongly linked to mental and physical health, wellbeing, and reduced mortality risk (Holt-Lunstad et al., 2010; Trudel-Fitzgerald et al., 2019; Holt-Lunstad, 2024), these benefits should not be attributed to alcohol itself. Among students and young adults, hazardous drinking is consistently associated with lower life satisfaction, poorer mental health, and greater loneliness (Sæther et al., 2019; Jensen et al., 2021). Any apparent health correlates of moderate drinking are therefore more plausibly explained by social connection and selection effects than by ethanol exposure (Holt-Lunstad et al., 2010; Sudhinaraset et al., 2016; Sæther et al., 2019).

Drinking norms are also changing. In many high-income countries, youth drinking has declined and alcohol-free forms of socialization are becoming more common (Törrönen et al., 2019; Whitaker et al., 2023; Livingston et al., 2024). Although abstinence can still create social friction in strongly drinking-oriented settings, it is increasingly compatible with social integration, particularly among younger and educated groups (Morris et al., 2020; Chok et al., 2024; Livingston et al., 2024).

This review integrates the biochemistry and physiology of alcohol metabolism with its broader health, behavioral, and societal consequences. We discuss inter-individual and population-level variability in alcohol disposition, alcohol’s effects on cognition and disease risk, the possible role of endogenous ethanol-acetaldehyde systems in cross-species stress resilience, and methodological challenges in alcohol research, particularly the use of heterogeneous non-drinker comparison groups. We close by considering changing drinking norms and the increasing acceptability of low-alcohol and abstinent lifestyles in contemporary societies.

Several recent reviews have already addressed complementary aspects of this broad field, including alcohol-driven oxidative metabolism and redox injury, oxidative stress and autophagy in alcohol-mediated brain damage, and related molecular pathways in multi-organ and reproductive toxicity (Kong et al., 2024; Ruiter-Lopez et al., 2025; Maccioni et al., 2026). These contributions are acknowledged as important parallel syntheses, while the distinctive aim of the present review is to connect these mechanistic frameworks with inter-individual genetic and epigenetic variability, behavioral consequences of ethanol-derived metabolites, emerging therapeutic approaches, beverage composition, and the methodological interpretation of non-drinker comparison groups.

## 2 Biochemical pathways and cellular consequences of alcohol metabolism

### 2.1 Enzymatic pathways of ethanol oxidation

Alcohol metabolism is a coordinated biochemical process that determines both the rate of ethanol clearance and the degree to which biologically reactive metabolites are generated. After ingestion, ethanol is metabolized predominantly in the liver. The first and quantitatively most important step is catalyzed by alcohol dehydrogenase (ADH), which oxidizes ethanol to acetaldehyde (Figure 1A). Acetaldehyde is a highly reactive intermediate and is therefore rapidly converted to acetate by aldehyde dehydrogenase (ALDH), mainly the mitochondrial isoenzyme *ALDH2* (Lieber, 2004; Crabb et al., 2004; Zakhari, 2006; Edenberg, 2007; Cederbaum, 2012; Wilson and Matschinsky, 2020; Haseba, 2025; Liu et al., 2025). Acetate is subsequently activated to acetyl-CoA, a central metabolic intermediate that enters energy production, lipid metabolism, amino-acid metabolism, and biosynthetic pathways (Riveros-Rosas et al., 1997; Bose and Locasale, 2019).

**FIGURE 1:**
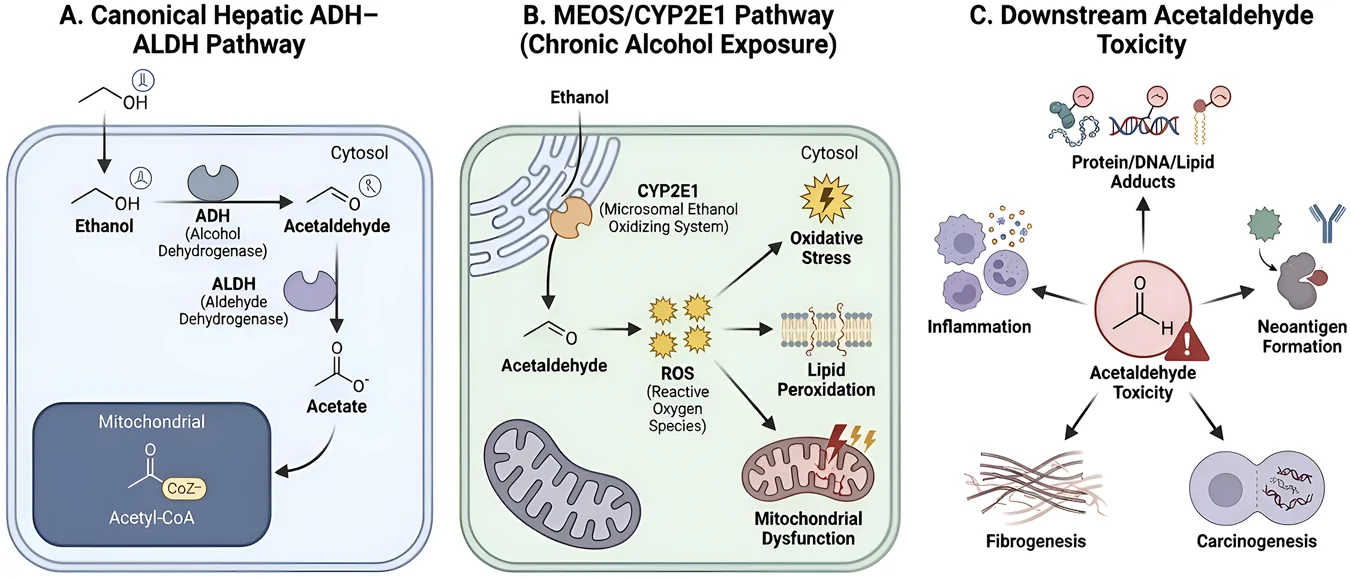
Integrated pathways of ethanol metabolism and acetaldehyde toxicity. **(A)** In the canonical hepatic alcohol dehydrogenase-aldehyde dehydrogenase pathway, ethanol is oxidized primarily by alcohol dehydrogenase (ADH) to acetaldehyde, a highly reactive and toxic intermediate. Acetaldehyde is subsequently converted by aldehyde dehydrogenase, mainly mitochondrial *ALDH2*, to acetate, which can be activated to acetyl-CoA and enter central metabolic pathways. **(B)** During chronic alcohol exposure or high ethanol concentrations, the microsomal ethanol-oxidizing system contributes increasingly to ethanol metabolism. This pathway is mediated mainly by CYP2E1, which oxidizes ethanol to acetaldehyde while promoting reactive oxygen species (ROS) generation. CYP2E1 induction thereby amplifies oxidative stress, lipid peroxidation, mitochondrial dysfunction, and inflammatory liver injury. **(C)** Acetaldehyde toxicity results from covalent modification of proteins, DNA, and lipids, leading to enzyme dysfunction, impaired DNA repair, altered cellular structure, neoantigen formation, immune activation, inflammation, fibrogenesis, and carcinogenesis. Together, these pathways explain how alcohol metabolism functions not only as a clearance mechanism but also as a source of biologically reactive intermediates that drive tissue injury.

At higher ethanol concentrations and during chronic alcohol exposure, the microsomal ethanol-oxidizing system (MEOS) contributes more substantially to ethanol oxidation (Figure 1B). MEOS is mediated primarily by cytochrome P450 2E1 (CYP2E1), which converts ethanol to acetaldehyde and is strongly inducible by repeated alcohol intake (Lieber, 1997; Albano, 2006; Zakhari, 2006; Lu and Cederbaum, 2008; Cederbaum, 2012; Massart et al., 2022). CYP2E1 induction is biologically important because it increases the generation of reactive oxygen species (ROS), thereby promoting oxidative stress, lipid peroxidation, mitochondrial dysfunction, inflammation, and progressive liver injury (Ronis et al., 1996; Dey and Cederbaum, 2006; Lu et al., 2008; Ceni et al., 2014; Hyun et al., 2021). Because CYP2E1 also participates in the metabolism of many xenobiotics and drugs, chronic alcohol exposure may alter pharmacokinetics and increase the risk of clinically relevant alcohol-drug interactions (Lieber, 1997; Zakhari, 2006; Cederbaum, 2012).

### 2.2 Acetaldehyde toxicity, adduct formation, and immune recognition

Acetaldehyde is one of the principal mediators of alcohol-related toxicity. It forms covalent adducts with proteins, lipids, and DNA, thereby interfering with enzyme activity, cytoskeletal organization, mitochondrial function, DNA repair, redox balance, and immune recognition (Tuma, 2003; Seitz and Stickel, 2007; Setshedi et al., 2010; Hayashi et al., 2013; Mizumoto et al., 2017). These effects contribute to hepatocellular injury, inflammation, fibrogenesis, and carcinogenesis (Figure 1C), particularly in tissues exposed to high local acetaldehyde concentrations such as the upper aerodigestive tract (Seitz and Stickel, 2007; Brooks et al., 2009; Chang et al., 2017; Im et al., 2021; Liu et al., 2025). Acetaldehyde-modified proteins can also act as neoantigens, stimulating immune responses that may further amplify tissue injury (Israel et al., 1986; Niemelä et al., 1987; Tuma, 2003; Setshedi et al., 2010).

### 2.3 Endogenous ethanol and acetaldehyde

Ethanol and acetaldehyde are not exclusively derived from alcoholic beverages. Endogenous ethanol has been detected in humans and animals, and arises from microbial fermentation, host metabolic reactions, and disease-associated changes in gut microbial activity (Ostrovsky, 1986; Jones, 1994; Al-Awadhi et al., 2004; Meijnikman et al., 2024; Stamation et al., 2025). In microorganisms and plants, ethanol and acetaldehyde are produced through conserved fermentative pathways, especially under hypoxic or anaerobic conditions (Kimmerer and MacDonald, 1987; Bui et al., 2019). Reported basal blood concentrations of endogenous ethanol and acetaldehyde in humans are approximately 100 μM and 1 μM, respectively, although these values vary widely depending on analytical method, physiological state, microbiome activity, and disease state (Ostrovsky, 1986; Jones, 1994; Meijnikman et al., 2024). Their physiological roles in mammals are likely context-dependent and remain incompletely defined (Ostrovsky, 1986; Zakhari, 2006; Meijnikman et al., 2024); proposed functions include modulation of membrane viscosity, neuroregulation, modulation of mitochondrial uncoupling, and immunomodulation (Ostrovsky, 1986; Meijnikman et al., 2024).

### 2.4 Acetaldehyde, central nervous system, and salsolinol

Ethanol readily crosses the blood-brain barrier and can be locally converted to acetaldehyde within the brain, mainly through catalase- and CYP2E1-dependent pathways; ADH appears to play a less prominent role in most brain regions (Zimatkin et al., 2006; Deng and Deitrich, 2008; Peana et al., 2017). Peripheral acetaldehyde, by contrast, does not freely enter the brain under physiological conditions because aldehyde-metabolizing systems at the blood-brain barrier limit its passage; nevertheless, brain acetaldehyde may rise after high ethanol exposure, with impaired ALDH activity, or following local ethanol oxidation (Isse et al., 2005; Peana et al., 2017). Experimental studies link brain acetaldehyde to oxidative stress, altered dopamine metabolism, neuroinflammation, neurobehavioral changes, and impaired learning (Heap et al., 1995; Foddai et al., 2004; Quertemont et al., 2005; Peana et al., 2017).

Behavioral evidence indicates that acetaldehyde is not merely a toxic by-product but can act as a neuroactive mediator whose effects depend on dose, route of administration, brain region, and metabolic context. In animal models, direct or metabolically enhanced acetaldehyde exposure has been associated with locomotor stimulation, conditioned reinforcement and place preference, voluntary intake, and modulation of anxiety- and stress-related responses, whereas high systemic exposure can produce aversion, sedation, motor impairment, and malaise. Conversely, pharmacological sequestration of acetaldehyde or inhibition of its formation can attenuate several ethanol-induced locomotor and motivational responses. Together, these findings support a contribution of brain-derived acetaldehyde to selected behavioral effects of ethanol, while also emphasizing that ethanol itself and other metabolites remain active and that extrapolation from intracranial animal paradigms to human drinking behavior requires caution (Correa et al., 2012; Peana et al., 2017).

Acetaldehyde may also influence neurochemistry through condensation reactions with biogenic amines. Its non-enzymatic Pictet-Spengler condensation with dopamine generates salsolinol and related tetrahydroisoquinolines, which have been implicated in ethanol reinforcement, mesolimbic dopamine signaling, and µ-opioid-receptor-related pathways (Figure 2A) (Hipólito et al., 2012; Polache and Granero, 2013; Israel et al., 2015; Berrios-Carcamo et al., 2017; Deehan et al., 2013; Peana et al., 2017; Bassareo et al., 2021). Acetaldehyde-derived β-carbolines can also form through reactions with serotonin-derived precursors (Figure 2A) (Quertemont et al., 2005; Peana et al., 2017). Endogenous mammalian morphine biosynthesis, however, is more commonly linked to dopamine-derived tetrahydropapaveroline-norlaudanosoline intermediates rather than to salsolinol as a direct precursor (Figure 2A) (Poeaknapo et al., 2004; Boettcher et al., 2005; Laux-Biehlmann et al., 2013). Acetaldehyde-derived amine condensates are therefore best described as neuroactive compounds with opioid-related and reward-modulating properties rather than as definitive “morphine-like neurotransmitters” (Quertemont et al., 2005; Peana et al., 2017). The accumulation of acetaldehyde also inhibits the oxidative deamination of dopamine, leading to elevated 3,4-dihydroxyphenylacetaldehyde levels (Figure 2A) (Peana et al., 2017).

**FIGURE 2:**
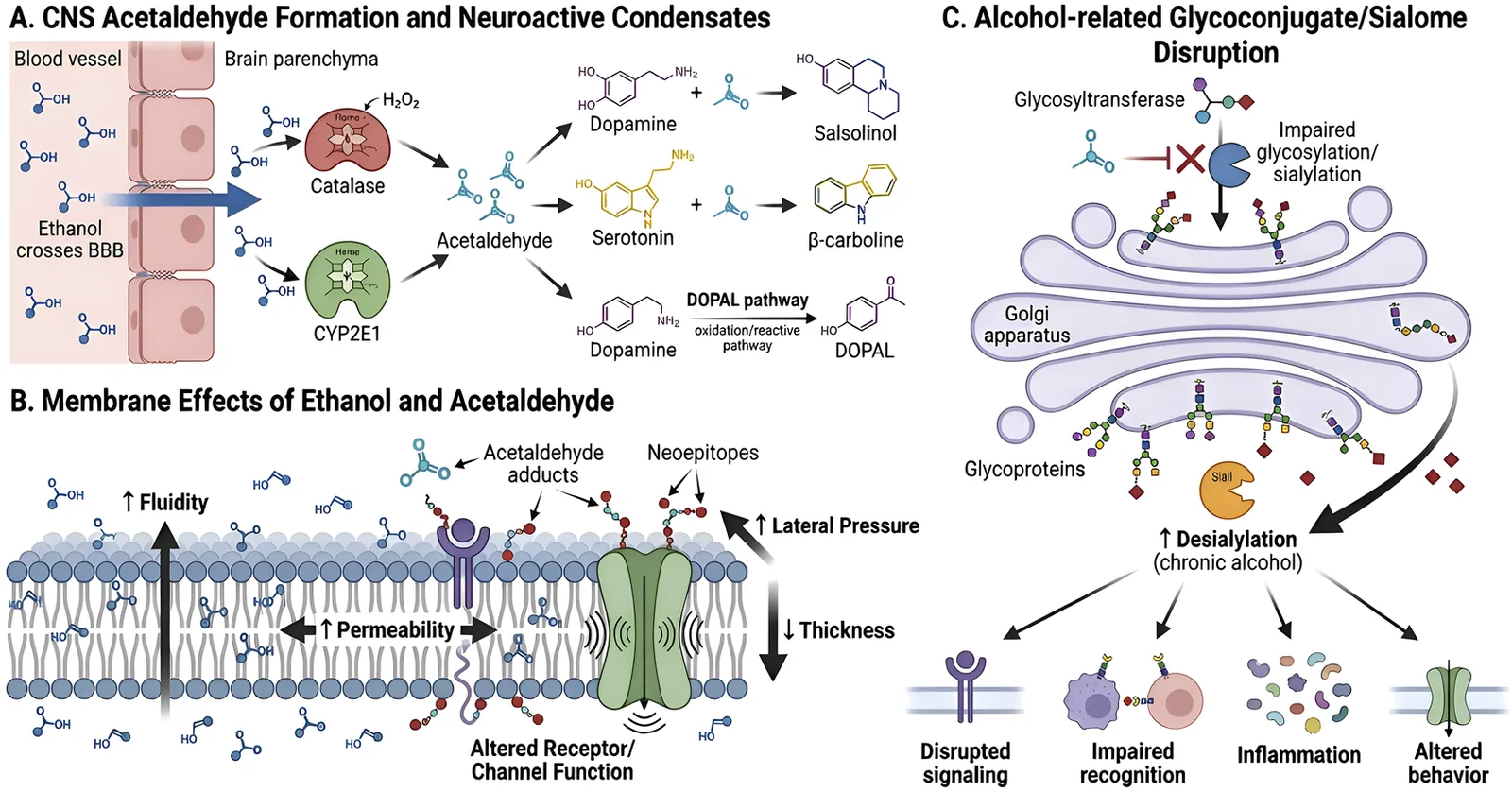
Neurochemical and membrane-level effects of ethanol and acetaldehyde. **(A)** ethanol readily crosses the blood-brain barrier and can be locally metabolized in the central nervous system, mainly through catalase- and CYP2E1-dependent pathways, producing acetaldehyde within brain regions involved in reward and behavior. Acetaldehyde can react with dopamine to form salsolinol and related tetrahydroisoquinolines, and with serotonin-derived precursors to form beta-carbolines, thereby influencing mesolimbic dopamine signaling, reinforcement, and opioid-related pathways. Acetaldehyde accumulation may also alter dopamine catabolism, including the dopamine oxidation/DOPAL pathway. **(B)** At the membrane level, ethanol partitions into lipid bilayers and alters membrane fluidity, permeability, thickness, elasticity, lateral pressure, and domain organization. These biophysical changes can modify receptor and ion-channel conformation, clustering, trafficking, and activity. In parallel, acetaldehyde forms covalent adducts with membrane and cytosolic proteins, potentially impairing protein function and generating neoepitopes recognized by the immune system. **(C)** Alcohol exposure also disrupts glycoconjugate and sialome homeostasis by impairing Golgi-dependent glycosylation/sialylation and increasing sialidase-mediated desialylation. The resulting imbalance in sialylation and desialylation affects cell-surface charge, receptor signaling, immune recognition, inflammation, membrane-complex stability, and ion-channel behavior.

Salsolinol provides a more specific link between acetaldehyde metabolism and reward circuitry. Experimental work in the posterior ventral tegmental area indicates that salsolinol can activate dopaminergic neurons, increase dopamine output in the nucleus accumbens shell, and engage mu-opioid-receptor-dependent mechanisms that promote reinforcement and behavioral sensitization (Hipólito et al., 2012; Polache and Granero, 2013; Bassareo et al., 2021). Bassareo and colleagues further demonstrated ethanol-dependent formation of salsolinol in the posterior ventral tegmental area and linked this local synthesis to mesolimbic dopamine activation (Bassareo et al., 2021). Nevertheless, the quantitative contribution of salsolinol to ethanol responses remains debated because its concentrations are low, analytical measurements are technically difficult, dietary and endogenous sources may contribute, and effects vary by stereoisomer, exposure pattern, and brain region (Hipólito et al., 2012; Peana et al., 2017).

### 2.5 Membrane biology and glycoconjugate metabolism

Ethanol partitions into lipid bilayers and acutely alters membrane fluidity, permeability, thickness, elasticity, lateral pressure, and domain organization. These changes can modify the conformation, clustering, trafficking, and activity of membrane-associated proteins, including receptors and ion channels (Figure 2B) (Chin and Goldstein, 1977; Goldstein, 1986; Crews et al., 1996; Patra et al., 2006; Harris et al., 2008; Lundbaek et al., 2010; Ingolfsson and Andersen, 2011). Acetaldehyde, in parallel, forms covalent adducts with membrane and cytosolic proteins, altering protein function and generating neoantigenic epitopes (Figure 2B) (Gaines et al., 1977; Israel et al., 1986; Barry et al., 1987; Tuma, 2003; Hayashi et al., 2013).

Beyond direct adduct formation, ethanol metabolism and acetaldehyde exposure perturb the metabolism of cellular glycoconjugates by disrupting the balance between sialylation and desialylation. Acetaldehyde inhibits hepatic glycoprotein synthesis and secretion at the level of terminal Golgi-dependent glycosylation, an effect demonstrable both in liver slices and in cultured hepatocytes (Sorrell and Tuma, 1978; Marinari et al., 1993). In parallel, alcohol exposure increases sialidase-mediated removal of N-acetylneuraminic acid from the non-reducing ends of glycoprotein and glycolipid glycans in the cell-surface glycocalyx (Figure 2C), resulting in the accumulation of hyposialylated and asialylated glycoconjugates and consequent functional impairment of the corresponding membrane complexes (Azuine et al., 2005; Garige et al., 2006; Chrostek et al., 2006; O’Flaherty et al., 2022; Zhu et al., 2024). Because terminal sialic acids regulate cell-surface charge, glycoprotein stability, cell recognition, immune interactions, and ion-channel function, altered sialylation can secondarily affect receptor signaling, transmembrane transport, inflammation, and immune tolerance (Klemm, 1987; Gong et al., 2007; Varki and Varki, 2007; Ednie and Bennett, 2012; Waszkiewicz et al., 2012; Schnaar et al., 2014; Zhu et al., 2024). The specific contribution of alcohol-induced sialome changes to stress resilience, cognitive maturation, autoimmunity, and pharmacoresistance remains an emerging mechanistically plausible hypothesis rather than a firmly established phenomenon (Mahajan and Pillai, 2016; Lübbers et al., 2018; Jastrząb et al., 2023).

To summarize, alcohol metabolism should not be viewed simply as a detoxification pathway. It generates reactive intermediates that affect mitochondrial function, redox balance, membrane organization, glycoconjugate metabolism, immune recognition, neural signaling, and tissue injury. These interconnected mechanisms help explain why alcohol exposure produces diverse biological effects across organs and individuals.

### 2.6 Factors affecting alcohol metabolism

The rate and efficiency of alcohol metabolism vary substantially between individuals, contributing to differences in alcohol tolerance, susceptibility to intoxication, and long-term health risk (Table 1) (Ramchandani et al., 2001; Zakhari, 2006; Edenberg and McClintick, 2018). The principal determinants are genetic and epigenetic, namely, polymorphisms and regulatory changes in genes encoding the alcohol-metabolizing enzymes, but they operate against a background of physiological modifiers including sex, age, body composition, chronic alcohol exposure, comorbidities, and concomitant medications (Zakhari, 2006; Edenberg and McClintick, 2018; Pathak and Pandey, 2017; Zhou and Gelernter, 2024; Meijnikman et al., 2024).

**TABLE 1:** Factors affecting alcohol metabolism.

Factor	Description	Impact on alcohol metabolism
Genetic variation	Polymorphisms in ADH and ALDH (e.g., *ADH1B rs1229984, ALDH2 rs671*) alter enzyme activity	Faster or slower acetaldehyde clearance; influences AUD risk, toxicity, and disease susceptibility
Sex	Lower gastric ADH and higher body-fat percentage in women	Higher peak BAC for equivalent doses; faster intoxication and greater long-term vulnerability
Age	Reduced liver function and total body water with aging	Higher BAC and prolonged effects in older individuals
Chronic alcohol use	Induction of CYP2E1 and the MEOS pathway	Increased acetaldehyde and ROS; tolerance, hepatotoxicity, drug interactions
Diet and nutrition	Deficiencies in zinc, thiamine, and other micronutrients	Impaired enzyme activity; greater oxidative stress and inflammation
Drug interactions	Co-administration of substrates of hepatic P450 enzymes (e.g., acetaminophen)	Altered metabolism of either alcohol or drug; increased hepatotoxicity
Liver disease	Hepatitis, cirrhosis, MASLD, MetALD.	Reduced metabolic capacity; greater systemic toxicity

### 2.6.1 Genetic and epigenetic variation in ADH and ALDH

Functional variants in alcohol dehydrogenase (ADH) and aldehyde dehydrogenase (ALDH) genes are the most extensively characterized molecular determinants of inter-individual differences in alcohol disposition (Table 2) (Yoshida et al., 1984; Bosron and Li, 1986; Crabb et al., 2004; Edenberg, 2007; Edenberg and McClintick, 2018). The two best-characterized variants are ADH1B*2 (rs1229984), which encodes a higher-activity ADH and accelerates ethanol oxidation to acetaldehyde, and ALDH2*2 (rs671), which markedly reduces the activity of the mitochondrial ALDH and impairs acetaldehyde clearance (Yoshida et al., 1984; Crabb et al., 1989; Peng and Yin, 2009; Chen et al., 1999; Edenberg and McClintick, 2018). In carriers of either variant, and especially in those who carry both, even moderate drinking can produce transient hyperacetaldehydaemia and the characteristic alcohol-flushing reaction, with facial flushing, nausea, headache, dizziness, and tachycardia (Crabb et al., 1989; Brooks et al., 2009; Chang et al., 2017). The aversive nature of this response is itself protective: meta-analyses in East Asian populations consistently show that ADH1B*2 and ALDH2*2 are each associated with substantially reduced risk of alcohol use disorder, with odds ratios of approximately 0.22–0.49 for ADH1B*2 and 0.22–0.25 for ALDH2*2, and with synergistic effects when the two protective alleles co-occur (Luczak et al., 2006; Chen et al., 1999; Zaso et al., 2019; Quillen et al., 2014). Conversely, in carriers of impaired *ALDH2* who continue to drink, sustained acetaldehyde exposure markedly increases the risk of alcohol-related cancers, particularly squamous-cell carcinoma of the oesophagus and other cancers of the upper aerodigestive tract (Seitz and Stickel, 2007; Yang et al., 2010; Brooks et al., 2009; Chang et al., 2017; Chang et al., 2023; Im et al., 2021; Liu et al., 2025). *ALDH2* deficiency has also been linked to cardiovascular consequences of even light-to-moderate drinking, including increased susceptibility to ventricular arrhythmia (Lee et al., 2023).

**TABLE 2:** Genes associated with alcohol metabolism.

Gene	Function	Significant polymorphism	References
*ADH1B*	Encodes alcohol dehydrogenase; oxidizes ethanol to acetaldehyde. Polymorphisms influence enzyme activity and ethanol elimination	rs1229984 (Arg47His): increased enzyme activity; reduced risk of alcohol dependence	Edenberg, 2007
*ADH1C*	Encodes alcohol dehydrogenase; influences ethanol metabolism and BAC.	rs698 (Ile349Val): Reduced elimination efficiency; higher risk of dependence	Hurley and Edenberg, 2012
*ALDH2*	Encodes aldehyde dehydrogenase; oxidizes acetaldehyde to acetate	rs671 (Glu504Lys): Markedly reduced activity; acetaldehyde accumulation; protective against AUD via aversion	Yoshida et al., 1984
*CYP2E1*	Microsomal ethanol-oxidizing system; contributes to ROS production	rs3813867: alters enzyme inducibility and oxidative stress	Zakhari, 2006
*GABRA2*	GABA-A receptor subunit; modulates inhibitory neurotransmission	rs279858: linked to AUD and anxiety-related traits	Enoch et al., 2006
*SLC6A3*	Dopamine transporter; reward and addiction circuitry	rs27072: associated with alcohol-withdrawal seizures in alcohol-dependent patients	Le Strat et al., 2008
DRD2	Dopamine D2 receptor; reward processing	rs1800497 (Taq1A): altered receptor density; addiction risk	Blum et al., 1990
CHRNA5	Nicotinic acetylcholine-receptor subunit; addiction-related behaviors	rs16969968: associated with alcohol dependence	Wang et al., 2009

The behavioral consequences of these variants extend beyond pharmacokinetics. Rapid acetaldehyde generation or impaired clearance produces an immediate aversive phenotype that can reduce initiation, escalation, and persistence of drinking, whereas individuals without this protective response may experience fewer acute deterrents. The same genotype can therefore lower AUD risk through behavioral avoidance yet increase cancer and organ-toxicity risk among carriers who continue to consume. These opposing effects illustrate why genetic associations should be interpreted jointly with drinking pattern, cultural context, sex, age, and comorbidity rather than as simple deterministic predictors (Hendershot et al., 2010; Edenberg and McClintick, 2018; Gross et al., 2015).

Beyond coding-sequence polymorphisms, epigenetic regulation adds a dynamic layer that can both precede and result from alcohol exposure. Acute and chronic ethanol exposure can alter DNA methylation, histone acetylation and methylation, chromatin accessibility, and non-coding RNA expression in liver and brain, thereby modifying genes involved in ADH, ALDH, and CYP2E1 expression, oxidative stress, neuroplasticity, inflammation, and reward signaling (Zakhari, 2013; Berkel and Pandey, 2017; Pathak and Pandey, 2017; Peana et al., 2017; Liu et al., 2023; Zhou and Gelernter, 2024). Some alcohol-associated epigenetic changes may persist during abstinence and contribute to craving, stress sensitivity, or relapse vulnerability. However, directionality is often uncertain because tissue specificity, cellular composition, nutrition, smoking, medication use, and prior drinking history can confound human epigenetic studies. Evidence for stable transgenerational inheritance in humans remains insufficient; accordingly, epigenetic markers are currently more useful as mechanistic and exposure-related candidates than as established clinical predictors.

Epigenetic mechanisms may also provide an important link between the immediate biochemical effects of alcohol and the development of persistent disease phenotypes. Thus, acetaldehyde and reactive oxygen species can disrupt one-carbon metabolism and interfere with the enzymes that regulate DNA methylation and histone modifications (Berkel and Pandey, 2017). At the same time, alcohol-responsive microRNAs may alter gene networks involved in antioxidant defense, inflammation, neuronal differentiation, synaptic plasticity, and stress responses (Terracina et al., 2024). During development, epigenetic changes induced within critical exposure windows may persist after alcohol has been cleared and contribute to long-term neurodevelopmental impairment (Sessa et al., 2025). In adolescents and adults, repeated alcohol exposure may also reinforce neuroadaptations associated with cognitive dysfunction, affective symptoms, craving, and relapse (Berkel and Pandey, 2017; Terracina et al., 2024). These mechanisms offer a plausible explanation for how acute metabolic injury can contribute to longer-term neuropsychiatric and systemic consequences. However, tissue specificity, reversibility, and uncertainty regarding causal direction remain important limitations of current human studies.

### 2.6.2 Population-level distribution of ADH/ALDH variants

The protective alleles described above are unevenly distributed between populations. The ALDH2*2 allele is largely restricted to East Asian populations, where its frequency typically ranges from 0.11 to 0.28; it is essentially absent in Europeans and Africans (Goedde et al., 1992; Wall et al., 2016; Zaso et al., 2019). ADH1B*2 is also most common in East Asians but is found at appreciable frequencies in some Middle-Eastern and Jewish populations as well (Goedde et al., 1992; Edenberg, 2007; Edenberg and McClintick, 2018). Surveys of ADH/ALDH isoenzyme phenotypes and genotypes in English, Chinese, Native American, Mexican-American, and other populations have helped to map this variation and to relate it to alcohol-sensitivity and alcohol-dependence phenotypes (Ricciardi et al., 1983; Rex et al., 1985; Bosron et al., 1988; Goedde et al., 1992; Eng et al., 2007; Ehlers et al., 2012; Schaschl et al., 2022). A summary of these distributions, with stratification by age group where available, is provided in Table 3 (Eng et al., 2007; Ehlers et al., 2012).

**TABLE 3:** Population distribution of protective *ALDH2* and *ADH1B* alleles.

Population/group	Age group or cohort information	ALDH2*2 distribution	ADH1B*2 distribution	Phenotype/interpretation	Key sources
East Asian populations (overall)	General population; age stratification not specified in the supplied paragraph	Largely restricted to East Asian populations; typical allele frequency approximately 0.11–0.28	Most common in East Asians	ALDH2*2 is linked to alcohol-sensitivity responses (e.g., flushing) and lower habitual drinking; ADH1B*2 is generally treated as protective against alcohol-dependence phenotypes	Goedde et al., 1992; Wall et al., 2016; Zaso et al., 2019; Edenberg, 2007; Edenberg and McClintick, 2018
Chinese, Chinese-American, Han Chinese, and Taiwanese groups	Population-level Asian literature review; no age strata stated in the supplied paragraph	Among the Asian groups in which ALDH2*2 appears most prevalent	High prevalence reported across Asian groups in literature reviews	Contributes to well-described alcohol-sensitivity variation in East Asian populations	Eng et al., 2007; Goedde et al., 1992
Japanese and Korean groups	Population-level Asian literature review; no age strata stated in the supplied paragraph	Among the Asian groups in which ALDH2*2 appears most prevalent	High prevalence reported across Asian groups in literature reviews	*ALDH2* genotype is repeatedly related to alcohol sensitivity and drinking-behavior phenotypes	Eng et al., 2007; Wall et al., 2016
Other Asian groups (Thai, Filipino, Indian, Chinese/Taiwanese aboriginal groups)	Population-level Asian literature review; no age strata stated in the supplied paragraph	Lower rates reported than in Chinese, Japanese, Korean, and Taiwanese groups	Generally high in Asians, but lower/exceptional patterns noted for Indians	Illustrates heterogeneity within Asia and cautions against treating “Asian” as a single genetic category	Eng et al., 2007
Middle-Eastern populations	Age stratification NR.	NR in the supplied paragraph	Appreciable frequencies reported in some Middle-Eastern populations	May contribute to between-population variation in alcohol sensitivity and alcohol-dependence risk	Goedde et al., 1992; Edenberg, 2007; Edenberg and McClintick, 2018
Jewish populations	Age stratification NR.	NR in the supplied paragraph	Appreciable frequencies reported in some Jewish populations	Potential contributor to population differences in alcohol-related phenotypes	Goedde et al., 1992; Edenberg, 2007; Edenberg and McClintick, 2018
Europeans/English populations	English populations included in ADH/ALDH phenotype and genotype surveys; age stratification NR.	Essentially absent in europeans	NR in the supplied paragraph	Used as comparison populations in surveys mapping alcohol-metabolism variation	Ricciardi et al., 1983; Rex et al., 1985; Bosron et al., 1988; Goedde et al., 1992
Africans	Age stratification NR.	Essentially absent in Africans	NR in the supplied paragraph	Used as comparison populations in global ADH/ALDH surveys	Goedde et al., 1992; Wall et al., 2016; Zaso et al., 2019
Native americans	Community sample of Native americans living on reservations (n = 791). Demographic variables including age were tested, but allele variants were not associated with age in the cited study	No Native American participants possessed an ALDH2*2 allele in Ehlers et al., 2012	At least one ADH1B*2 allele found in 7% of participants	ADH1B*2 was not associated with protection against alcohol dependence in this Native American sample; ADH1B*3, a related *ADH1B* protective variant, was associated with protection	Ehlers et al., 2012
Mexican americans	Community sample of Mexican americans living in the same county as the Native American sample (n = 391). Demographic variables including age were tested, but allele variants were not associated with age in the cited study	Two Mexican American participants possessed one ALDH2*2 allele in Ehlers et al., 2012	At least one ADH1B*2 allele found in 13% of participants	ADH1B*2 was associated with protection against alcohol dependence in this Mexican American sample	Ehlers et al., 2012
Other surveyed populations	Varies by study; age stratification NR in the supplied paragraph	NR in the supplied paragraph	NR in the supplied paragraph	ADH/ALDH isoenzyme phenotype and genotype surveys helped map between-population variation and relate it to alcohol-sensitivity and alcohol-dependence phenotypes	Schaschl et al., 2022; Goedde et al., 1992

Abbreviations: NR, not reported in the supplied source paragraph; ADH2*2 is the older nomenclature for ADH1B*2.

The general principle that emerges from these studies is consistent: efficient acetaldehyde generation combined with impaired acetaldehyde clearance produces aversive reactions that discourage heavy drinking and reduce dependence risk, whereas continued drinking under the same conditions amplifies acetaldehyde-mediated toxicity and disease risk (Chen et al., 1999; Peng and Yin, 2009; Brooks et al., 2009; Chang et al., 2017; Edenberg and McClintick, 2018). (Earlier reports describing an “18-fold lower frequency of ADH2 phenotypes among non-dependent than among alcohol-dependent individuals” used now-obsolete ADH2/ADH2*2 nomenclature corresponding to ADH1B/ADH1B*2; modern studies more typically express the same biology as a protective effect of the high-activity ADH1B*2 allele rather than as a deficit in the low-activity variant (Stamatoyannopoulos et al., 1975; Borras et al., 2000; Yokoyama and Omori, 2003; Edenberg and McClintick, 2018).)

It should also be noted that genetic effects do not act in isolation: even within East Asian populations carrying the same protective genotypes, mean alcohol consumption can vary substantially across cultural and geographic contexts, illustrating the importance of gene–environment interaction (Millwood et al., 2019). Because acetaldehyde is a major mediator of alcohol-related somatic injury and contributes to several neurobehavioral effects of ethanol (Tuma, 2003; Quertemont et al., 2005; Setshedi et al., 2010), the population-level distribution of ADH and ALDH alleles helps to explain part, but not all, of the variation in alcohol sensitivity and disease risk observed across populations.

Reported alcohol intake is only an indirect measure of the internal toxic burden experienced by an individual. Thus, high-activity variants of *ADH1B* and *ADH1C* may accelerate the oxidation of ethanol to acetaldehyde, whereas reduced *ALDH2* activity can impair acetaldehyde clearance and prolong tissue exposure (Edenberg and McClintick, 2018). Increased *CYP2E1* expression or inducibility may further enhance acetaldehyde generation and production of reactive oxygen species, particularly during repeated or chronic alcohol exposure (Cederbaum, 2012). Genetic variation in catalase, glutathione metabolism, DNA repair, and other antioxidant or detoxification pathways may additionally modify the cellular response to alcohol-derived metabolites (Sessa et al., 2025). Individual susceptibility therefore reflects the balance between metabolite formation, clearance, and cellular defence, which together determine peak acetaldehyde concentrations, exposure duration, adduct formation, oxidative injury, and organ-specific damage. This framework supports genotype- and phenotype-informed risk assessment and more precise stratification of research cohorts. However, current evidence does not justify the use of any single genetic variant to define an individually safe level of alcohol consumption.

### 2.6.3 Sex differences

Sex is a robust modifier of alcohol pharmacokinetics. After equivalent doses, women generally reach higher peak blood alcohol concentrations (BACs) than men, primarily because of (i) lower gastric ADH activity and consequently reduced first-pass metabolism, (ii) smaller total body water and lower lean body mass, which result in a smaller volume of distribution, and (iii) related differences in liver volume and hepatic clearance (Frezza et al., 1990; Thomasson, 1995; Mumenthaler et al., 1999; Ely et al., 1999; Baraona et al., 2001; Vatsalya et al., 2023). These pharmacokinetic differences contribute to faster onset of intoxication in women and to greater long-term vulnerability to alcohol-related liver disease, cardiovascular disease, and certain cancers at lower exposure thresholds than in men (Baraona et al., 2001; Wilsnack et al., 2013; Thibaut, 2023; Paton, 2005). Reproductive-hormone fluctuations across the menstrual cycle do not appear to substantially affect alcohol pharmacokinetics (Mumenthaler et al., 1999), although hormonal status, pregnancy, and menopause may modulate tissue-specific susceptibility and warrant further study (Thibaut, 2023).

### 2.6.4 Age

Aging is associated with progressive changes that increase the impact of a given alcohol dose. Total body water and lean body mass decline, the volume of distribution for ethanol decreases, and hepatic mass and blood flow are reduced; the activities of ethanol- and acetaldehyde-metabolizing enzymes (ADH, ALDH, and CYP2E1) also tend to diminish (Vestal et al., 1977; Pozzato et al., 1995; Seitz et al., 2004; Meier and Seitz, 2008; Mancinelli and Vitali, 2004; Brick and Erickson, 2009). The combined effect is higher peak BACs and prolonged exposure at any given intake, with greater central-nervous-system sensitivity, slower recovery from acute intoxication, and increased risk of hepatic and other organ injury in older drinkers (Liangpunsakul et al., 2010a; Liangpunsakul et al., 2010b; White and Hingson, 2013). Older adults also tend to be on more concurrent medications, increasing the risk of clinically significant alcohol–drug interactions, particularly with central-nervous-system depressants and substrates of hepatic CYP enzymes (Sorock et al., 2006; Meier and Seitz, 2008).

### 2.6.5 Chronic alcohol consumption and CYP2E1 induction

Habitual alcohol intake itself reshapes alcohol metabolism. Repeated exposure induces CYP2E1 and increases the contribution of the microsomal ethanol-oxidizing system (MEOS) to ethanol clearance (Lieber, 1997; Zakhari, 2006; Lu and Cederbaum, 2008; Cederbaum, 2012; Massart et al., 2022). This induction has two important consequences. First, by increasing the rate of ethanol oxidation, it contributes to the development of metabolic tolerance, which can lead drinkers to consume larger quantities to achieve the same subjective effect—and consequently to greater overall exposure to acetaldehyde and other toxic intermediates (Arteel, 2003; Zakhari, 2006; Cederbaum, 2012). Second, induced CYP2E1 is itself a major source of reactive oxygen species (ROS) and reactive nitrogen species, generating a state of sustained oxidative stress that drives lipid peroxidation, mitochondrial dysfunction, hepatocellular injury, and inflammation (Albano, 2006; Dey and Cederbaum, 2006; Lu et al., 2008; Cederbaum, 2012). Cumulatively, these effects underlie the histological progression from steatosis through steatohepatitis to fibrosis, cirrhosis, and hepatocellular carcinoma in chronic heavy drinkers (Arteel, 2003; Seitz and Stickel, 2007; Hyun et al., 2021; Liu et al., 2025). CYP2E1 also metabolizes a wide range of xenobiotics, so chronic drinkers are more susceptible to clinically relevant alcohol–drug interactions and to drug-induced hepatotoxicity, exemplified by potentiated paracetamol toxicity (Lieber, 1997; Cederbaum, 2012).

### 2.6.6 Diet, nutrition, and concomitant medications

Nutritional status modulates alcohol metabolism and its consequences. Deficiencies of zinc, thiamine, folate, methionine, and other micronutrients impair the activity or stability of alcohol-metabolizing enzymes and exacerbate oxidative stress; protein-energy malnutrition further compromises hepatic regeneration and detoxification capacity (Lieber, 2003; McClain et al., 2002). Concomitant intake of substrates or inhibitors of hepatic cytochrome P450 enzymes, including paracetamol, certain antimicrobials, and many psychoactive medications, can alter the metabolism of alcohol or the medication and increase the risk of toxicity (Lieber, 1997; Weathermon and Crabb, 1999; Cederbaum, 2012). Pre-existing liver disease, obesity, type-2 diabetes, and metabolic dysfunction-associated steatotic liver disease (MASLD) all reduce the liver’s reserve capacity to metabolize alcohol and increase susceptibility to alcohol-induced injury, a constellation now formally recognized in the MetALD category of the 2023 multi-society nomenclature for steatotic liver disease (Arab et al., 2025; Israelsen et al., 2025).

### 2.7 Toward genotype- and phenotype-informed prevention of alcohol abuse

Inefficient acetaldehyde clearance - most often a consequence of reduced *ALDH2* activity but also achievable through other pharmacological or pathological routes - produces a state of chronic acetaldehyde exposure with genotoxic, carcinogenic, and pro-inflammatory consequences (Yoshida et al., 1984; Brooks and Theruvathu, 2005; Albano, 2006; Seitz and Stickel, 2007; Brooks and Zakhari, 2013). Combined with the oxidative stress generated by induced *CYP2E1*, this acetaldehyde exposure drives the inflammatory, fibrogenic, and carcinogenic cascades described above and is a major mechanism underlying both alcohol-associated liver disease and cancers of the upper aerodigestive tract (Matsuo et al., 2006; Peng and Yin, 2009; Seitz and Becker, 2007; Liu et al., 2025).

These observations support the case for personalized approaches to alcohol-related risk communication and prevention. Genetic screening for *ADH1B* and *ALDH2* variants, already feasible in research settings, could in principle identify individuals at elevated risk of alcohol-related cancer or organ injury and enable more informative counselling, while pharmacological strategies aimed at modulating ADH/ALDH activity or scavenging acetaldehyde represent active areas of therapeutic development (Edenberg and Foroud, 2013; Brooks and Zakhari, 2013; Owaki et al., 2024; Liu et al., 2025). Translation into routine practice will require carefully designed prospective trials in which behavioral and psychosocial factors that co-vary with metabolic phenotype are explicitly addressed, since differences in genotype and metabolism are typically intertwined with differences in drinking behavior, comorbidity, and dependence risk (Hendershot et al., 2010; Edenberg and Foroud, 2013; Gross et al., 2015).

### 2.8 Prenatal exposure: Maternal-fetal asymmetry and epigenetic programming

Pregnancy provides a particularly clear example of why alcohol-related harm cannot be inferred from maternal intake alone. Primarily, ethanol and acetaldehyde can expose the placental-fetal compartment, while the developing fetus has limited ADH and ALDH activity and comparatively weak antioxidant defences (Terracina et al., 2024). Maternal variants in *ADH1B*, *ADH1C*, *ALDH2*, *CYP2E1*, and related detoxification pathways may influence the magnitude and duration of fetal exposure to ethanol, acetaldehyde, and reactive oxygen species. At the same time, fetal genotype and placental function may further modify local metabolism, detoxification capacity, and tissue susceptibility to ethanol and acetaldehyde (Sessa et al., 2025). This maternal-fetal metabolic asymmetry can be additionally shaped by nutritional status, smoking, medication use, metabolic disease, psychosocial stress, and the developmental timing of exposure. Finally, alcohol-induced alterations in DNA methylation, histone regulation, and microRNA expression in placental and fetal tissues may further reprogram gene expression and contribute to persistent neurodevelopmental and neuropsychiatric consequences (Terracina et al., 2024; Sessa et al., 2025).

These mechanisms may help explain the marked phenotypic variability observed in fetal alcohol spectrum disorders despite apparently similar reported levels of prenatal alcohol exposure. However, currently available molecular biomarkers remain investigational and cannot replace primary prevention or comprehensive clinical and developmental assessment (Sessa et al., 2025). Fetal alcohol spectrum disorders should therefore be understood as the outcome of interactions among alcohol exposure, maternal and fetal genotype, placental biology, and environmental context, rather than as a uniform consequence of maternal dose alone.

## 3 Alcohol and cognitive health

Conventional epidemiological studies have long reported a J-shaped association between alcohol intake and dementia risk, suggesting that light-to-moderate drinking might confer protection relative to either heavy drinking or abstention (Holahan et al., 2010; Topiwala and Ebmeier, 2018; Schwarzinger et al., 2018). The same pattern is observed for all-cause mortality and has been variously attributed to cardiovascular benefits, antioxidants in alcoholic beverages, or social-behavioral correlates of moderate drinking. However, observational designs cannot disentangle alcohol’s direct effects from differences between individuals who self-select into different drinking categories. A growing body of evidence indicates that the apparent benefits of light drinking are largely driven by selection: moderate drinkers tend to be more economically secure, better educated, healthier at baseline, and to drink in socially regulated contexts, while non-drinkers are over-represented for prior heavy drinking, ill-health, or other risk factors (Naimi et al., 2005; Stockwell et al., 2016; Naimi et al., 2017; Zhao et al., 2023; Sarich et al., 2024; Stockwell et al., 2024).

Mendelian randomization (MR) circumvents many of these confounders by using genetic variants associated with alcohol intake as instrumental variables for lifetime exposure (Topiwala et al., 2018; Millwood et al., 2019; Bouajila et al., 2024; Rosoff et al., 2025). In contrast to the J-shaped curve from observational studies, recent linear and non-linear MR analyses do not support a protective effect of light or moderate drinking on dementia risk and instead suggest a monotonic, dose-dependent increase in risk among current drinkers (Andrews et al., 2020; Zheng et al., 2024; Campbell et al., 2024). For example, Zheng and colleagues used a 95-SNP genetic instrument in 313,958 UK Biobank current drinkers and found that the J-shape evident in conventional analyses was not supported by non-linear MR, with their findings suggesting no safe level of alcohol consumption for cognitive health (Zheng et al., 2024). These results align with prior work showing alcohol-related reductions in brain volume and cognitive performance (Topiwala and Ebmeier, 2018; Larsson et al., 2014; Schwarzinger et al., 2018).

These findings have substantial public-health implications. If alcohol’s effect on cognition is approximately linear, then even moderate drinking carries some risk, and previous guidelines acknowledging a “safe” threshold may need revision (Stockwell et al., 2016; World Health Organization, 2023; Zheng et al., 2024). Given the irreversibility of dementia and its rising global incidence, the most cautious interpretation of current evidence is that minimal alcohol intake is preferable for protecting brain health in later life (Schwarzinger et al., 2018; Wiegmann et al., 2020; Bouajila et al., 2024; Zheng et al., 2024).

Mechanistic heterogeneity is an important consideration in the interpretation of population-based alcohol studies. Categories such as “one or two drinks per day” implicitly assume broadly comparable biological exposure, although the same reported intake may produce substantially different acetaldehyde kinetics and oxidative burdens depending on variation in ADH, ALDH, and CYP2E1 (Edenberg and McClintick, 2018). Epigenetic state, sex, age, body composition, hepatic function, metabolic health, and concurrent exposures may further modify the resulting internal toxic burden (Sessa et al., 2025). Population-level estimates may therefore combine individuals with relatively efficient ethanol metabolism and acetaldehyde clearance with those who experience prolonged metabolite exposure and greater oxidative injury. Such heterogeneity can obscure susceptible subgroups and weakens the biological basis for a universal J-shaped association or fixed safety threshold. The absence of a single safe level of alcohol consumption is therefore supported not only by bias-adjusted epidemiological analyses and Mendelian randomization studies (Millwood et al., 2019; Zheng et al., 2024), but also by mechanistic evidence showing that a given external dose does not translate into a uniform internal exposure across genetically and physiologically diverse populations.

## 4 Current advances in the treatment of alcohol-associated disorders

Therapeutic interventions for alcohol abuse and the associated disorders should be assessed according to the quality and maturity of the supporting evidence rather than their novelty. Current standard care for AUD combines structured psychosocial interventions with pharmacotherapy when clinically indicated. Among available medications, oral naltrexone and acamprosate are supported by the strongest evidence for reducing the risk of return to drinking, whereas disulfiram and several off-label agents are generally reserved for selected clinical situations (McPheeters et al., 2023). Accordingly, the therapeutic strategies discussed below are categorized by evidence level as established, adjunctive, emerging clinical, experimental in humans, or preclinical (Table 4).

**TABLE 4:** Evidence hierarchy and translational status of current and emerging interventions for alcohol use disorder.

Evidence category	Intervention examples	Current evidence base	Main limitations and translational barriers
Established standard	Structured psychosocial treatment; oral naltrexone; acamprosate	Multiple randomized controlled trials, systematic reviews, and meta-analyses support their use as first-line care	Contraindications, adherence, access to structured care, and the need for individualized treatment selection
Selected or context-dependent	Disulfiram; topiramate; gabapentin; baclofen	Disulfiram is established for selected supervised patients; evidence for off-label agents is variable and indication-dependent	Need for supervision, adverse effects, comorbidities, drug interactions, and heterogeneous patient selection
Adjunctive	Exercise and structured lifestyle interventions	Clinical trials and systematic reviews support their use as additions to pharmacological and psychosocial treatment	Adherence, non-standardized intervention intensity and outcomes, and insufficient evidence as stand-alone treatment
Emerging clinical	GLP-1 receptor agonists; ketamine-assisted therapy	Early randomized trials, small clinical studies, and observational evidence show promising effects on craving or alcohol consumption	Limited sample sizes, uncertain durability, active-comparator trials, long-term safety, cost, and patient selection
Experimental human	Low-intensity focused ultrasound neuromodulation	Small safety and feasibility studies in humans; efficacy for AUD remains unconfirmed	Target validation, sham-controlled trials, durability of response, scalability, and specialist delivery requirements
Preclinical	Catalytic gastrointestinal hydrogel for ethanol detoxification	Animal and mechanistic studies indicate reduced blood alcohol concentrations and tissue injury	Human toxicology and pharmacokinetics, microbiome and nutrient effects, reproducible manufacturing, and the risk of compensatory drinking
Regimen-specific	Combination pharmacotherapy, including naltrexone plus gabapentin	Selected trials support particular combinations, but there is no general class-wide effect	Optimal drug pairing, sequencing, dosing, interactions, biomarker selection, and confirmation against established standard care

Abbreviations: AUD, alcohol use disorder; GLP-1, glucagon-like peptide-1.

### 4.1 Current standard treatment

Oral naltrexone and acamprosate represent the principal evidence-supported pharmacotherapies for AUD and are typically combined with motivational, cognitive-behavioral, or other structured psychosocial interventions (McPheeters et al., 2023). Treatment selection can be individualized according to the intended therapeutic goal, whether abstinence or reduced drinking, and should also consider opioid use, hepatic and renal function, anticipated adherence, psychiatric comorbidities, and patient preference. For instance, disulfiram is more appropriate for selected, highly motivated patients, particularly when adherence can be supervised (McPheeters et al., 2023). At the same time, topiramate, gabapentin, and baclofen remain off-label or context-dependent alternatives whose use should be guided by the available evidence and individual clinical circumstances (Anton et al., 2011; Mason and Lehert, 2012). Emerging interventions should therefore be evaluated against these established standards of care rather than against the absence of treatment.

### 4.2 Catalytic gels for intra-luminal alcohol detoxification

Evidence level: preclinical. Su and colleagues recently described a single-site iron-anchored amyloid hydrogel that catalyzes the conversion of ethanol directly to acetic acid in the gastrointestinal tract, bypassing acetaldehyde (Su et al., 2024). In mice, oral administration reduced blood alcohol concentrations by up to ∼50% and attenuated alcohol-induced liver and gastrointestinal damage. By preventing acetaldehyde generation in the gut-portal axis, this approach could plausibly mitigate both acute intoxication and the chronic oxidative-inflammatory injury underlying alcohol-related liver disease (Ruan and Fast, 2008; Purohit et al., 2009). Translation to humans will require pharmacokinetic characterization, dose optimization, long-term safety assessment, and clinical trials measuring meaningful efficacy outcomes; nevertheless, this work illustrates a broader principle of catalytic biomolecular detoxification that may apply to other drug-related toxicities (Moore and Lippard, 2012).

### 4.3 GLP-1 receptor agonists

Evidence level: emerging clinical. Glucagon-like peptide-1 (GLP-1) receptor agonists (GLP-1 RAs) developed for type 2 diabetes and obesity, such as liraglutide and semaglutide, have shown robust effects on alcohol consumption in preclinical models and increasing evidence in humans (Egecioglu et al., 2013; Suchankova et al., 2015; Vallof et al., 2016; Klausen et al., 2022). GLP-1 receptors are expressed in mesolimbic structures involved in alcohol reinforcement, including the ventral tegmental area and nucleus accumbens, and GLP-1 RAs reduce alcohol-induced dopamine release and operant alcohol self-administration in rodents (Egecioglu et al., 2013; Vallof et al., 2016). In a recent phase 2 randomized clinical trial, once-weekly low-dose semaglutide reduced laboratory alcohol self-administration and weekly craving in adults with AUD compared to placebo, supporting the conduct of larger AUD-focused trials (Hendershot et al., 2025). Observational and pharmacoepidemiological studies have similarly reported reductions in AUD-related hospitalizations among GLP-1 RA users, although heterogeneity between agents and populations remains to be tested (Wium-Andersen et al., 2022; Lähteenvuo et al., 2025). Beyond their effects on ethanol consumption, these agents may also reduce hepatic and cardiovascular consequences of chronic alcohol abuse (Farr et al., 2016). The aforementioned findings justify larger AUD-specific trials, but optimal dose, durability, comparative effectiveness against naltrexone or acamprosate, adverse effects, access, and effects in patients without obesity or diabetes remain unresolved.

### 4.4 Ketamine-assisted therapy

Evidence level: early clinical. Ketamine, a non-competitive NMDA-receptor antagonist with rapid antidepressant effects, is being investigated as an adjunct to behavioral therapy for AUD (Krystal et al., 2013; Sahakian et al., 2010; Niciu and Sanacora, 2018; Williams and Heifets, 2019). The proposed mechanism involves enhanced neuroplasticity that may render addictive habits more amenable to revision through cognitive behavioral therapy or motivational enhancement (Krystal et al., 2013). Systematic reviews of available trials report reductions in craving, increased abstinence rates, and improvements in comorbid depression and anxiety, although evidence is limited by small sample sizes, methodological heterogeneity, and challenges with blinding given ketamine’s distinctive psychoactive effects (Grabski et al., 2022; Kelson et al., 2023). Optimization of dosing, scheduling, and integration with psychotherapy, as well as long-term safety, remain priorities for ongoing research. Further translation of ketamine therapy may require standardized psychotherapy, monitoring for dissociation, blood-pressure and misuse risks, as well as direct comparison with established AUD treatments.

### 4.5 Focused ultrasound neuromodulation

Evidence level: experimental human feasibility. Low-intensity focused ultrasound (LIFU) is a non-invasive neuromodulation technique that can target deep brain structures with millimeter precision, including the nucleus accumbens, a central node of the reward circuit (Hamani and Temel, 2012; Yoon et al., 2019; Blackmore et al., 2019). In a first-in-human safety and feasibility trial, LIFU targeting the bilateral nucleus accumbens in patients with substance use disorders was well tolerated and associated with reductions in self-reported craving over a 90-day follow-up (Mahoney et al., 2023; Olaitan et al., 2024). As a non-surgical technique with adjustable parameters, LIFU may offer a tailored intervention for AUD and other addictive disorders, potentially combinable with cognitive-behavioral or motivational therapies, although larger randomized trials are required before clinical recommendations can be made.

### 4.6 Exercise and lifestyle interventions

Evidence level: adjunctive clinical. Structured exercise including aerobic activity, resistance training, and mind-body practices such as yoga reduces craving, improves mood, and supports overall wellbeing in people with AUD (Brown et al., 2014; Giesen et al., 2015; Linke and Ussher, 2015; Stoutenberg et al., 2016). Mechanistically, exercise stimulates endorphin release, increases brain-derived neurotrophic factor (BDNF), and supports neuroplastic recovery from alcohol-related damage (Zschucke et al., 2012). Exercise also fosters routine, social connection, and self-efficacy serving as non-pharmacological factors strongly associated with successful long-term recovery. Exercise should therefore be considered an evidence-supported adjunct rather than a substitute for pharmacological or psychosocial treatment of AUD.

### 4.7 Combination pharmacotherapy

Evidence level: mixed and regimen-specific. Because AUD involves dysregulation of multiple neural systems - reward, stress, and impulse control - combinations of medications targeting complementary mechanisms have attracted increasing attention (Heilig et al., 2010; Anton et al., 2011; Mason and Lehert, 2012; Haass-Koffler et al., 2014; Ray and Bujarski, 2014; Koob and Mason, 2016). For instance, naltrexone (opioid antagonist) combined with gabapentin (anxiolytic, anticonvulsant) combination has shown additive efficacy for reducing drinking and withdrawal symptoms in some trials, however these results cannot be generalized to all combinations (Anton et al., 2011). Combination regimens are also being explored as a vehicle for personalization, with selection guided by genetic, psychiatric, and physiological profile. However, optimal pairings, sequencing, and dosing remain to be established in controlled studies.

To summarize, the treatment of AUD is evolving from a limited set of established medications toward a multidisciplinary and mechanism-based framework that integrates pharmacotherapy, psychosocial care, lifestyle interventions, and neuromodulation. However, these approaches should be interpreted according to the maturity and quality of the supporting evidence rather than their novelty. Structured psychosocial interventions combined with oral naltrexone or acamprosate remain the principal evidence-based standards of care, while disulfiram is an established option whose effectiveness depends strongly on adherence and supervision (McPheeters et al., 2023). Exercise and other structured lifestyle interventions may provide useful clinical support but should be regarded as adjuncts rather than substitutes for established treatment. Among emerging strategies, GLP-1 receptor agonists and ketamine-assisted therapy have produced encouraging early clinical findings, although larger and adequately controlled trials with longer follow-up and clinically relevant drinking outcomes are still required (Grabski et al., 2022; Hendershot et al., 2025). Low-intensity focused ultrasound remains at the safety and feasibility stage, and its efficacy, durability, optimal stimulation parameters, and appropriate patient population have not yet been established (Mahoney et al., 2023). Combination pharmacotherapy is supported by a plausible mechanistic rationale, but the available evidence is heterogeneous and individual regimens require separate validation (Anton et al., 2011; Koob and Mason, 2016). Catalytic gastrointestinal detoxification remains a preclinical strategy and should not currently be regarded as a treatment for AUD (Su et al., 2024). The successful translation of these approaches will depend on their ability to demonstrate clinically meaningful benefits beyond current pharmacological and psychosocial care. Important barriers include small and highly selected study populations, difficulties with blinding, uncertain durability of treatment effects, adverse effects and drug interactions, patient selection, scalability, cost, and regulatory requirements. A clear evidence hierarchy is therefore essential when comparing established therapies with emerging and experimental interventions.

## 5 The overlooked role of beverage composition: fructose-containing alcoholic drinks and liver disease

Despite extensive research on ethanol metabolism and its pathological consequences, a critical variable remains insufficiently addressed in most epidemiological and mechanistic studies: the composition of the alcoholic beverage itself. Most clinical and population-based studies classify alcohol exposure primarily according to total ethanol intake, largely overlooking the biochemical diversity of alcoholic beverages, including their carbohydrate content, particularly fructose. This omission may represent a significant confounding factor in studies of alcohol-associated liver disease, since many commonly consumed alcoholic beverages (beer, sweet wines, liqueurs, mixed drinks) contain appreciable amounts of simple sugars, including fructose or fructose-generating carbohydrates. Importantly, fructose is independently established as a potent driver of hepatic steatosis and metabolic dysfunction (Tappy and Le, 2010; Vos and Lavine, 2013; Song et al., 2016).

Fructose metabolism is predominantly hepatic and bypasses key regulatory steps in glycolysis, leading to unrestrained substrate flux into *de novo* lipogenesis, triglyceride accumulation, mitochondrial dysfunction, oxidative stress, and insulin resistance - hallmarks of metabolic-dysfunction-associated steatotic liver disease (MASLD, formerly NAFLD) (Stanhope et al., 2009; Rocha et al., 2017; Softic et al., 2016). Unlike glucose metabolism, fructose metabolism rapidly depletes intracellular ATP and increases uric acid production, further exacerbating oxidative stress and inflammatory signaling (Lanaspa et al., 2012; Keim and Havel, 2013). Large bodies of experimental and clinical evidence link high fructose intake, particularly from sugar-sweetened beverages, to fatty liver, metabolic syndrome, and cardiovascular risk (Malik et al., 2010; Bray, 2013; Chiu et al., 2014).

Fructose-containing alcoholic beverages may therefore represent a dual metabolic insult, combining ethanol’s hepatotoxicity (acetaldehyde production, redox imbalance, and oxidative stress) with fructose’s lipogenic and pro-inflammatory effects. Ethanol and fructose metabolism converge on multiple pathogenic pathways: increased NADH/NAD^+^ ratio and altered redox balance (Lieber, 2004; Softic et al., 2016); enhanced *de novo* lipogenesis (Stanhope et al., 2009); impaired fatty-acid oxidation (Sanyal et al., 2001; Abdelmalek et al., 2010); mitochondrial dysfunction (Mantena et al., 2008; Lanaspa et al., 2012); ROS generation (Cederbaum, 2012; Rocha et al., 2017); and activation of inflammatory cascades (Szabo and Bala, 2010; Tilg and Moschen, 2010). Consistent with shared mechanisms, combined ethanol and fructose exposure exacerbates hepatic steatosis, inflammation, and fibrosis more than either substrate alone (Zhong et al., 2012; Kanuri et al., 2014). Mechanistically, ethanol may enhance intestinal permeability and endotoxin translocation (Szabo and Bala, 2010), while fructose amplifies lipogenesis and uric-acid-mediated oxidative stress (Johnson et al., 2013), together accelerating liver injury.

These observations raise an important and largely unexplored possibility: a significant fraction of liver damage attributed to alcohol *per se* may, in fact, be mediated or substantially amplified by concomitant fructose intake. In epidemiological studies in which beverage composition is not controlled, this can lead to overestimation of ethanol-specific toxicity and underappreciation of dietary co-factors. Conversely, ethanol may amplify fructose toxicity. From a bioenergetic standpoint, ethanol provides “empty calories” that do not contribute to metabolic regulation but profoundly alter hepatic redox state. By increasing the NADH/NAD^+^ ratio, ethanol suppresses fatty-acid oxidation and promotes lipid accumulation (Lieber, 2004; Cederbaum, 2012), potentiating the lipogenic effects of fructose. Ethanol-induced mitochondrial dysfunction may further impair the liver’s capacity to manage fructose-derived metabolic flux, creating a feed-forward loop of metabolic stress (Mantena et al., 2008).

This conceptual framework suggests that alcohol-related and metabolic fatty liver diseases are not entirely distinct entities but may represent overlapping manifestations of a shared metabolic disturbance driven by substrate overload and redox imbalance. The recently introduced category of MetALD (“metabolic dysfunction-associated steatotic liver disease with increased alcohol intake”) within the 2023 multi-society nomenclature update for steatotic liver disease formalizes this overlap and acknowledges patients in whom both metabolic and alcohol-related drivers are clinically active (Eslam et al., 2020; Targher et al., 2021; Arab et al., 2025; Israelsen et al., 2025). This unifying view has important implications for both research and clinical practice. Future studies should (i) stratify alcohol exposure not only by quantity but also by beverage composition, particularly sugar and fructose content; (ii) re-evaluate epidemiological associations between alcohol and liver disease after adjustment for dietary sugar intake; and (iii) investigate combined ethanol-fructose exposure in controlled experimental and clinical settings. From a public-health perspective, these insights suggest that reducing fructose intake, especially from sweetened alcoholic beverages, may be as important as limiting ethanol consumption in preventing liver disease.

In summary, the current paradigm attributing liver cirrhosis predominantly to ethanol toxicity is incomplete. Fructose, a well-established driver of fatty liver and metabolic syndrome, may act as a critical cofactor, and, in some contexts, a primary trigger of liver pathology traditionally classified as alcohol-induced. Recognizing this interaction can refine our understanding of liver disease etiology and supports an integrated metabolic model of liver injury (Figure 3) (Arab et al., 2025; Israelsen et al., 2025).

**FIGURE 3:**
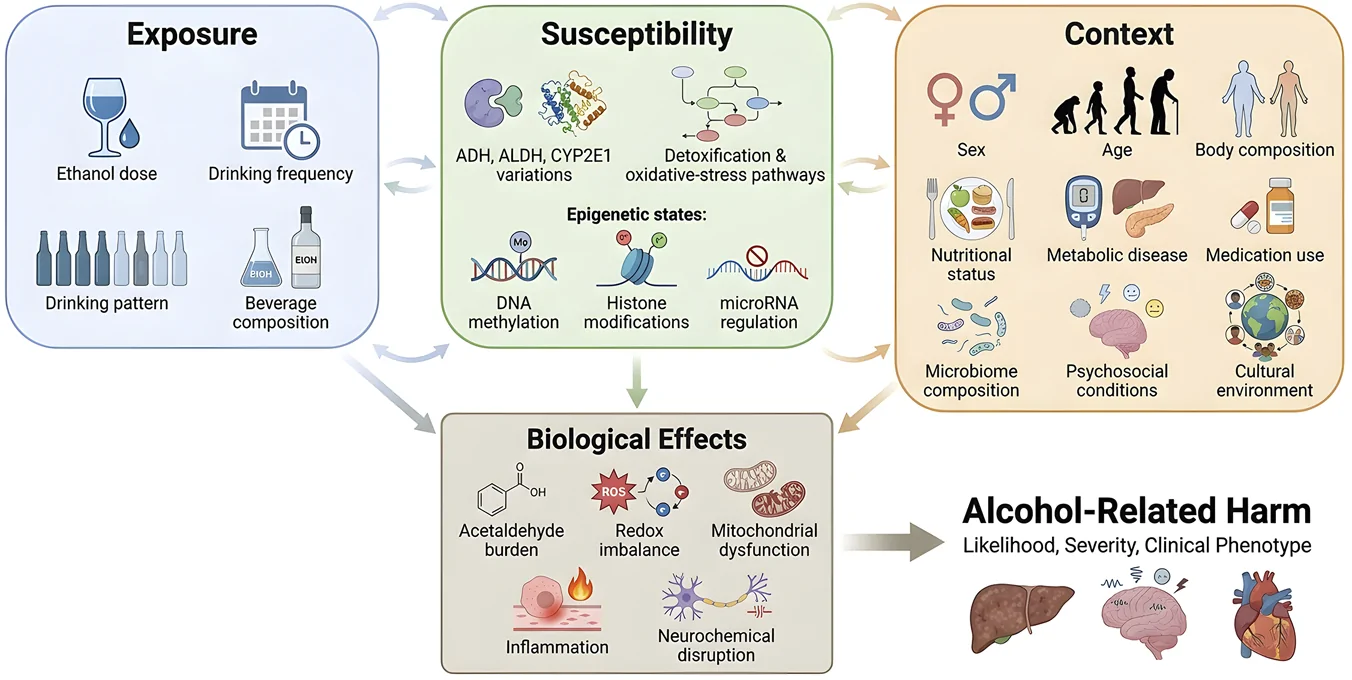
Exposure-susceptibility-context model of alcohol-related harm. Alcohol-related outcomes arise from the interaction among three principal domains: exposure, biological susceptibility, and physiological or environmental context. Exposure includes ethanol dose, drinking frequency and pattern, duration of use, and beverage composition. Susceptibility reflects genetic variation in alcohol-metabolizing and detoxification pathways, including ADH, ALDH, and CYP2E1, as well as differences in antioxidant defence, DNA repair, and epigenetic regulation through DNA methylation, histone modifications, and microRNAs. Context includes sex, age, developmental stage, body composition, nutritional status, metabolic disease, medication use, microbiome composition, psychosocial conditions, and cultural or social environment. These domains jointly determine the internal biological response to alcohol, including acetaldehyde accumulation, oxidative stress and redox imbalance, mitochondrial dysfunction, inflammation and immune activation, neurochemical disruption, and impaired DNA and cellular repair. Their combined effects generate heterogeneous clinical outcomes, including alcohol use disorder, liver and metabolic disease, neurological and neuropsychiatric impairment, cardiovascular complications, cancer, and fetal or developmental abnormalities. The feedback pathway indicates that established disease and its treatment may subsequently modify physiological context, susceptibility, and future alcohol-related risk. The model emphasizes that the same reported alcohol intake may produce substantially different internal toxic burdens and clinical consequences across individuals.

## 6 Resveratrol: reevaluating the scientific evidence behind the marketing claims

Resveratrol is a phytoalexin and trans-stilbene-derived polyphenol synthesized by several plants in response to fungal infection, injury, UV exposure, and other biotic and abiotic stresses (Langcake and Pryce, 1976; Adrian et al., 1996; Hasan and Bae, 2017). It is found in grape skin, peanuts, berries, and other plant-derived foods, and has been detected in red wine; reported concentrations vary widely, with commonly cited values around 0.2–5.8 mg/L and population means near 1.9 mg/L, while white wines generally contain substantially less (Stervbo et al., 2007; Prasad, 2012; Fabjanowicz et al., 2018).

Studies in mice and rats have demonstrated multiple biological effects of resveratrol - anti-inflammatory, antitumor, hypoglycemic, and cardioprotective - although these findings are largely preclinical and dose-dependent (Jang et al., 1997; Baur et al., 2006; Salehi et al., 2018). Resveratrol was once hailed as a “miracle molecule” with broad health benefits, including anti-aging effects, on the basis of early reports of sirtuin pathway activation and lifespan extension in model organisms (Howitz et al., 2003; Wood et al., 2004; Baur et al., 2006; Pezzuto, 2018). Lifespan extension was reported in yeast and certain invertebrate models and later in the short-lived fish *Nothobranchius furzeri* (Howitz et al., 2003; Wood et al., 2004; Valenzano et al., 2006). However, these findings were not consistently reproduced in *Drosophila melanogaster* and *Caenorhabditis elegans*, and later mechanistic studies questioned whether resveratrol directly activates SIRT1 under physiologically relevant conditions (Kaeberlein et al., 2005; Bass et al., 2007; Beher et al., 2009; Pacholec et al., 2010). Although many human trials have examined resveratrol for metabolic, cardiovascular, inflammatory, oncologic, or cognitive endpoints, no clinical trial has validated a lifespan-extending effect in humans (Smoliga et al., 2011; Ramirez-Garza et al., 2018; Brown et al., 2024).

Subsequent research has revealed substantial gaps in the evidence base (Smoliga et al., 2011; Novelle et al., 2015; Ramirez-Garza et al., 2018; Brown et al., 2024). Several issues are worth highlighting:

- Bioavailability. Resveratrol has poor systemic bioavailability in humans. Although oral absorption is relatively high, rapid first-pass conjugation produces glucuronide and sulfate metabolites and very low circulating concentrations of unchanged parent compound (Walle et al., 2004; Walle, 2011; Kapetanovic et al., 2011; Sergides et al., 2016; Springer and Moco, 2019). Some metabolites may have biological activity, but this complicates the interpretation of supplement efficacy (Walle, 2011; Springer and Moco, 2019).
- Dose discrepancies. Effective doses in rodents far exceed plausible human exposure from red wine or ordinary diet (Baur et al., 2006; Stervbo et al., 2007; Smoliga and Blanchard, 2014). Achieving rodent-study-equivalent exposures via wine would require impractical and harmful amounts of alcohol (Stervbo et al., 2007; Prasad, 2012; World Health Organization, 2023).
- Inconsistent clinical results. Human trials have produced mixed or modest findings. Some report metabolic improvements in selected groups (e.g., obese men), while others find no significant effects on insulin sensitivity or broad metabolic outcomes (Timmers et al., 2011; Poulsen et al., 2013; de Ligt et al., 2015). Reviews of human intervention studies conclude that resveratrol may produce context-specific effects on cardiometabolic, inflammatory, or vascular biomarkers, but the evidence is heterogeneous and insufficient for broad clinical recommendations (Ramirez-Garza et al., 2018; Brown et al., 2024; Godos et al., 2024).
- Lack of unique benefits. Many effects attributed to resveratrol including antioxidant and anti-inflammatory activity are shared by broader dietary patterns rich in fruits, vegetables, legumes, nuts, and other polyphenol-containing foods, and by physical activity and other lifestyle factors (Salehi et al., 2018; Brown et al., 2024). Resveratrol should therefore not be presented as a substitute for evidence-based lifestyle or medical interventions (World Health Organization, 2023; Brown et al., 2024).

The wellness industry’s narrative has often overlooked these limitations, particularly the gaps between preclinical mechanism, supplement dosing, bioavailability, and clinically meaningful outcomes (Novelle et al., 2015; Pezzuto, 2018; Brown et al., 2024). Marketing has often invoked red wine, luxury, longevity, and the “French paradox” despite the fact that alcohol itself is a toxic, dependence-producing substance and a Group 1 carcinogen, and red wine should not be recommended as a route to obtain resveratrol (Ronis, 1996; World Health Organization, 2023; Brown et al., 2024). Health claims commonly rely on selective interpretation of preclinical data or surrogate biomarkers rather than on robust evidence of reduced disease incidence, delayed aging, or extended human lifespan (Smoliga et al., 2011; Ramirez-Garza et al., 2018; Brown et al., 2024). Although resveratrol is not devoid of biological activity, its effects in humans remain far from the transformative promises suggested by early headlines (Novelle et al., 2015; Pezzuto, 2018; Brown et al., 2024). The molecule’s progression from scientific curiosity to overhyped health product illustrates the gap between preliminary mechanistic research and consumer expectations shaped by marketing. For improving health, a balanced dietary pattern, regular physical activity, avoidance of smoking, moderation or avoidance of alcohol, and evidence-based medical care remain more reliable than resveratrol supplementation (World Health Organization, 2023; Brown et al., 2024).

## 7 Validity and representativeness of non-drinker samples in alcohol research

A pervasive but under-recognized methodological problem in observational alcohol research is the heterogeneous composition of the “non-drinker” comparison group (Shaper et al., 1988; Naimi et al., 2017; Stockwell et al., 2024). Several distinct sub-populations are typically combined under this label, with very different health profiles. First, individuals with chronic illnesses such as liver disease, cardiovascular disorders, or metabolic conditions may reduce or stop drinking because of their condition, placing them at inherently higher risk of adverse outcomes independent of current alcohol intake (Park et al., 2017; Ng Fat et al., 2014; Yu et al., 2025). Second, some individuals carry genetic polymorphisms of alcohol-metabolizing enzymes (e.g., *ADH1B*, *ALDH2*) that produce physiological aversion to alcohol via flushing and other unpleasant reactions (Edenberg, 2007; Peng and Yin, 2009; Chang et al., 2017). Third, a substantial subset abstains for cultural, religious, lifestyle, or personal reasons, which may or may not correlate with other health-related behaviors (Epler et al., 2009; Kerr et al., 2017; Rosansky and Rosenberg, 2020; Yu et al., 2025). Pooling these subgroups into a single “non-drinker” category introduces heterogeneity, exposure misclassification, and confounding that can bias study results (Caldwell et al., 2006; Rehm et al., 2008; Stockwell et al., 2016; Naimi et al., 2017).

A particularly important bias is the so-called “sick quitter” effect, in which individuals who previously drank cease drinking because of declining health or alcohol-related harm (Shaper et al., 1988; Ng Fat et al., 2014; Park et al., 2017). This group is often misclassified as part of the current non-drinker population and inflates the apparent harm associated with abstention relative to moderate drinking (Rehm et al., 2008; Stockwell et al., 2016; John et al., 2021; Yu et al., 2025). Failure to separate former drinkers and sick quitters from lifelong abstainers can create the misleading impression that moderate alcohol consumption confers cardiovascular and longevity benefits (Naimi et al., 2005; Naimi et al., 2017; Zhao et al., 2023; Sarich et al., 2024; Stockwell et al., 2024). Lifelong abstainers, when accurately identified, represent a more appropriate reference group; however, they are often underrepresented or inconsistently classified (Caldwell et al., 2006; Ng Fat et al., 2014; Ekholm et al., 2024).

These biases have substantial implications for the validity of studies of alcohol consumption and AUD (Rehm et al., 2008; Crane et al., 2017; Carr et al., 2024). If the non-drinker group is disproportionately composed of individuals with pre-existing illness, prior heavy drinking, or AUD, the apparent benefits of moderate drinking are overestimated (Fillmore et al., 2007; Ng Fat et al., 2014; John et al., 2021). Conversely, the true risks of alcohol may be obscured by inclusion of non-drinkers with poor baseline health (Shaper et al., 1988; Park et al., 2017; Sarich et al., 2024).

To improve validity, several methodological adjustments are recommended:

- Stratification of non-drinkers into lifelong abstainers, former drinkers (sick quitters), and individuals abstaining for cultural or religious reasons.
- Use of lifelong abstainers as the primary reference group, where feasible.
- Incorporation of genetic data (e.g., *ADH1B* and *ALDH2* variants) to identify individuals who avoid alcohol because of physiological aversion.
- Longitudinal designs that distinguish pre-existing conditions from the effects of current alcohol consumption.

Without appropriate stratification, estimates of the risks and potential benefits associated with alcohol consumption may be substantially biased. Lifelong abstainers, former drinkers, individuals with genetically mediated alcohol aversion, and those who abstain for cultural or religious reasons may differ considerably in baseline health, prior alcohol exposure, metabolic phenotype, and underlying susceptibility. Reliable biological interpretation of population-level associations therefore requires validated classification of abstainer subgroups, adjustment for baseline health and prior drinking history, longitudinal follow-up, and, where appropriate, genetic or metabolic stratification.

## 8 Proposed exposure-susceptibility-context model

To integrate the evidence reviewed in this article, we propose an exposure-susceptibility-context model of alcohol-related harm (Figure 3). According to this model, exposure encompasses ethanol dose, frequency, drinking pattern, duration and beverage composition. Susceptibility reflects variation in ADH, ALDH, CYP2E1, and other pathways involved in detoxification and oxidative-stress responses, together with epigenetic states shaped by DNA methylation, histone modifications, and microRNA regulation. Context includes sex, age, body composition, nutritional status, metabolic disease, medication use, microbiome composition, psychosocial conditions, and cultural environment. These three domains interact to determine acetaldehyde burden, redox imbalance, mitochondrial dysfunction, inflammation, and neurochemical disruption, which together shape the likelihood, severity, and clinical phenotype of alcohol-related harm.

The model explains why alcohol exposure alone cannot determine individual risk or support a universal safe threshold. The same reported intake may produce substantially different internal acetaldehyde burdens and biological effects depending on an individual’s metabolic susceptibility and physiological or environmental context. Pregnancy represents a particularly important example because maternal and fetal alcohol metabolism are asymmetric, while alcohol-induced epigenetic reprogramming may contribute to persistent neurodevelopmental abnormalities and the long-term burden of fetal alcohol spectrum disorders (Sessa et al., 2022; Sessa et al., 2025). This framework supports more precise risk stratification, genotype- and phenotype-informed counselling, and the selection of preventive or therapeutic interventions according to the predominant drivers of risk in each individual. Finally, heterogeneity within non-drinker and comparison groups directly connects the epidemiological challenge to the proposed mechanistic framework, as these groups differ not only in current alcohol exposure but also in genetic background, prior tissue injury, physiological characteristics, and social context.

Operationally, the proposed framework distinguishes four interconnected levels: external alcohol exposure, internal ethanol and acetaldehyde burden, molecular and cellular responses, and clinical outcomes. Integrating conventional assessments of alcohol consumption with genetic, epigenetic, metabolic, and environmental data would enable mechanistic studies and clinical trials to identify individuals at increased risk and to define subgroups most likely to benefit from specific interventions, rather than relying solely on broad population-level exposure categories. Within this framework, the immediate contribution of precision medicine lies in improving prevention, risk stratification, and treatment selection, rather than in establishing genotype-specific thresholds for safe alcohol consumption. Complete avoidance of alcohol during pregnancy and reduction of alcohol exposure across the general population remain the most reliable preventive strategies.

Importantly, in the proposed model, fructose and other dietary components are metabolic cofactors rather than alternative explanations for ethanol toxicity. Their importance is likely greatest in individuals with obesity, insulin resistance, MASLD, or high consumption of sweetened alcoholic beverages, in whom substrate overload and redox imbalance converge. Future studies should quantify beverage composition and background diet so that ethanol-specific and combined effects can be separated. This interpretation is consistent with MetALD and avoids attributing all alcohol-associated liver injury either to ethanol alone or to fructose alone (Arab et al., 2025; Israelsen et al., 2025).

## 9 Concluding remarks

The biology of alcohol metabolism is intricate, generating not only the principal effects of acute intoxication but also the reactive intermediates and metabolic perturbations that drive its long-term consequences. Sex, age, body composition, nutrition, baseline health, and, critically -genetic and epigenetic variation in ADH, ALDH, and CYP2E1 jointly determine how each individual processes alcohol and how vulnerable they are to its harms. Recent advances in genetic epidemiology, particularly Mendelian randomization, have substantially weakened the long-standing inference that moderate drinking is protective against dementia and other chronic diseases, suggesting instead a monotonic relationship between alcohol intake and risk. At the same time, the formal recognition of MetALD highlights that alcohol- and metabolic-driven liver disease form a continuum rather than discrete entities, with beverage composition (fructose content) acting as an underappreciated modifier. Behavioral and neurochemical evidence further indicates that brain-derived acetaldehyde and salsolinol can participate in reinforcement and mesolimbic dopamine signaling, although their quantitative contribution in humans remains unresolved. Genetic variants and exposure-responsive epigenetic programs should therefore be viewed as interacting determinants of both drinking behavior and tissue vulnerability rather than as isolated metabolic modifiers.

The therapeutic landscape is also evolving. Catalytic gels that intercept ethanol before its conversion to acetaldehyde, GLP-1 receptor agonists that reduce craving and consumption, ketamine-assisted psychotherapy, focused-ultrasound neuromodulation, exercise-based interventions, and rationally designed combination pharmacotherapies all offer new avenues for treating AUD. Integrating these tools with genetically and behaviorally informed personalization, more rigorous methodological standards in observational research, particularly with respect to non-drinker comparison groups, and updated public-health communication that conveys current evidence on dose-risk relationships will be essential to reduce the global burden of alcohol-related disease.

The evidence reviewed in this article supports a unified framework in which alcohol-related disease results from the interaction between exposure, metabolic toxicity, biological susceptibility, and environmental context rather than from ethanol dose alone. The amount consumed and the pattern of drinking determine the initial exposure, whereas ADH, ALDH, CYP2E1, and tissue-specific metabolic pathways shape acetaldehyde accumulation and oxidative burden. Genetic and epigenetic variation, sex, age, pregnancy, organ functional reserve, and comorbid disease further influence individual susceptibility. Nutritional status, concomitant medications, beverage composition, and the social environment may additionally modify both exposure and biological response. These interacting factors converge on acetaldehyde-adduct formation, redox imbalance, mitochondrial dysfunction, inflammation, altered neurochemical signalling, and impaired cellular repair, thereby producing heterogeneous hepatic, neurological, carcinogenic, developmental, and behavioral outcomes. This framework explains why a universal threshold for risk-free alcohol consumption is biologically implausible and why reported benefits of moderate drinking remain highly susceptible to confounding, selection bias, and population averaging. It also outlines a precision-medicine agenda based on more accurate characterization of exposure, identification of high-risk metabolic and developmental contexts, prospective validation of genetic and epigenetic biomarkers, and alignment of established or emerging interventions with individual phenotype and the strength of the supporting evidence. Naltrexone, acamprosate, and structured psychosocial care remain the appropriate therapeutic benchmarks, whereas newer pharmacological, neuromodulatory, and metabolic strategies should be evaluated in comparative clinical trials. The practical objective is not to infer deterministic outcomes from genotype, but to identify modifiable points at which exposure reduction, targeted surveillance, or treatment can interrupt the progression from alcohol intake to disease.
